# The Role of Somatostatin Receptor Scintigraphy on the Diagnosis of Desmoid Tumors

**DOI:** 10.5402/2012/167545

**Published:** 2012-05-31

**Authors:** Joerg Friesenbichler, Anja Molcan, Reingard Aigner, Patrick Sadoghi, Bernadette Liegl-Atzwanger, Werner Maurer-Ertl, Christian Weger, Andreas Leithner

**Affiliations:** ^1^Department of Orthopedic Surgery, Medical University of Graz, Auenbruggerplatz 5, 8036 Graz, Austria; ^2^Division of Nuclear Medicine Radiology, Department of Radiology, Medical University of Graz, Auenbruggerplatz 9, 8036 Graz, Austria; ^3^Institute of Pathology, Medical University of Graz, Auenbruggerplatz 25, 8036 Graz, Austria

## Abstract

*Background*. Magnetic resonance imaging is considered as imaging modality of choice in diagnosis of desmoid tumors, though even this technique can lack the ability to distinguish aggressive fibromatosis from other benign or malignant soft tissue tumors. The aim of this study was to investigate if desmoid tumors would show an adequate tracer uptake in somatostatin receptor scintigraphy and moreover to correlate these results with immunohistochemical staining. *Patients and Methods*. Thirteen patients with desmoid tumors were examined with somatostatin receptor scintigraphy. Additionally, seven of these patients have been tested for the immunohistochemical expression of somatostatin receptor subtype 2A. The results of somatostatin receptor scintigraphy and the results of immunohistochemical staining (somatostatin receptor subtype 2A) were evaluated and correlated. *Results*. Somatostatin receptor scintigraphy revealed that eight of 13 affected patients (62%) showed an enhanced tracer uptake. On the other hand, the correlation between the results of somatostatin receptor scintigraphy and immunohistochemical investigations was poor (two out of seven cases). *Conclusion*. The current study demonstrated that desmoid tumors frequently express somatostatin receptor subtype 2, while immunohistochemical investigations did not correlate with these findings. This may likely be due to lack of standardization of this technique and also due to heterogeneous receptor distribution within the tumors.

## 1. Introduction

Fibromatoses/desmoid tumors are fibroblastic proliferations with an intermediate dignity between benign fibroblastic tumors and fibrosarcoma [1]. They are characterized by an infiltrative growth pattern, high rate of local recurrence, and the inability to metastasize [1–3]. Referring to Weiss and Goldblum [3] there are two main groups of fibromatoses- superficial (fascial) and deep (musculoaponeurotic) fibromatosis. Furthermore, these tumors are divided into several subcategories according to their anatomic location (abdominal, extra-abdominal, intra-abdominal). Wide or radical resection with negative microscopic surgical margins is treatment of choice and a significant prognostic factor in primary case as well as in recurrence cases (*P* < 0.001) [4].

MRI represents the gold-standard imaging modality for the purpose of planning management, especially surgery, and performing surveillance of desmoid patients in order to detect disease progression and postoperative recurrence [5–11].

Peptide receptor imaging (PRI) enables visualization of receptor expressing tissues noninvasively [12, 13]. Furthermore, peptides labeled with *β*-radiation emitters can be used to eradicate receptor expressing tissues (peptide receptor radionuclide therapy PRRT) [13].

However, the best examples of targets for radiopeptide-based imaging are somatostatin receptors (SSTRs), since they are frequently expressed in neuroendocrine tumors as well as in a wide variety of other malignancies, even soft tissue tumors [13, 14].

Studies on somatostatin receptor scintigraphy (SRS) have started in 1988 and have initially concerned with the use of 123I-Tyr3-octreotide, which has already been abandoned for practical reasons and cost concerns [15]. In 1994, imaging with the radiopharmaceutical 111In-pentetreotide (commercially available as OctreoScan) was approved for patients with neuroendocrine tumors (NETs) [13]. Positive scans reflect the presence of an increased density of SSTRs, in particular SSTR 2 and SSTR 5 [16]. Nevertheless, the density of SSTRs varies among different tumors and therefore, the sensitivity of 111In-pentetreotide also varies.

Therefore, new somatostatin analogues with similar binding profile and imaging quality but labeled with 99 mTc have frequently been studied and are growing in importance [12, 13, 17], due to the cost effectiveness and wide availability of this radioisotope [13, 18]. This somatostatin analogue shows high affinity to SSTR 2 and lowest to SSTR 5 [19]. Studies of various authors revealed several advantages of 99 mTc-TOC compared to 111In-OCT, hence 99mTc-TOC is considered as a promising radiopharmaceutical to replace 111In-OCT in diagnostic nuclear medicine [18, 20].

Although advanced imaging modalities can provide clues to the correct diagnosis of aggressive fibromatosis, histological examination of tumour tissue remains the golden diagnostic standard [5, 7, 9, 21].

Based on the report of de Pas et al. [22], the aim of this retrospective study was to investigate the percentage of desmoid tumors that express SSTR 2 and show adequate tracer uptake in SRS. The hypotheses were that there is a low percentage of SSTR 2 positive desmoid tumors and that only few cases show adequate uptake in SRS.

## 2. Materials and Methods

Thirteen patients who have been treated for desmoid tumors which have been histologically verified and who have been examined with SRS since 1999 were included in the current (Table 1). Gender distribution was balanced with six males and seven females. Mean age at diagnosis was 38 years (range, 18 to 52) and the average followup was 54 months (range, 6 to 153). Ten patients presented local recurrence and three patients had their second relapse.

Most tumors occurred in typical anatomical regions. Although deep fibromatoses are rare on hands and feet, one patient developed a metacarpal tumour. Another patient, who initially presented with a proliferation of the lower leg, developed a plantar recurrence. The extra-abdominal tumors were located at the following anatomical sites—trunk: *n* = 1; upper and lower extremities: *n* = 10, whereas six occurred in the upper extremity and four in the lower one.

All patients were analyzed either for uptake of 111In-DTPA-octreotide or for uptake of 99mTc-EDDA/HYNIC-TOC. Furthermore, scintigraphic findings were correlated with corresponding MR images as well as with immunohistochemical expression of SSTR 2A. Immunohistochemical staining and data analysis were performed whenever tissue material was available.

### 2.1. Somatostatin Receptor Scintigraphy (SRS)

**Figure 1:**
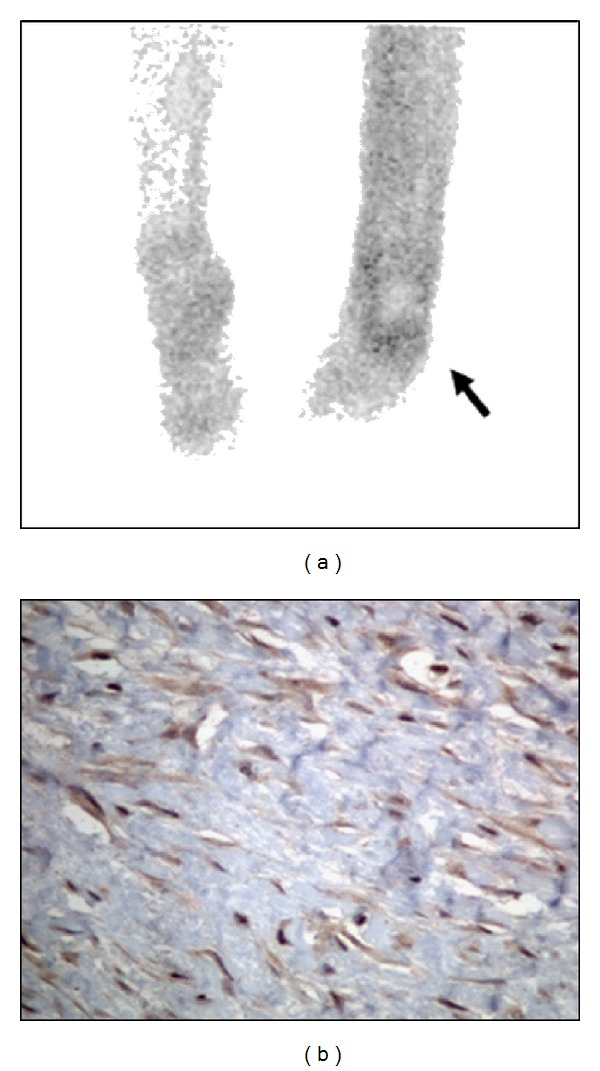
Positive correlation of 111In-DTPA-octreotide scintigraphy and somatostatin receptor 2A immunohistochemistry. (a) Results of SRS showing enhanced tracer uptake at the side of the desmoid tumour at the bottom of the foot and (b) moderate immunohistochemical staining for somatostatin receptor subtype 2A (magnification ×40).

Somatostatin receptor scintigraphy was performed either preoperatively or at suspicion of disease relapse, by using established radiopharmaceuticals, namely, either 111In-DTPA-octreotide or 99mTc-EDDA/HYNIC-TOC (Figure 1(a)). The tracers were prepared with commercially available kits using standard techniques as described by the producers. 111In-DTPA-octreotide was used as tracer until 2008. Planar and SPECT investigations of the tumour regions were performed 4 or 5 and 24 hours after application of the radioactive tracer. Since 2009 SRS has been performed with 99mTc-EDDA/HYNIC-TOC, due to cost effectiveness and a simplifying one-day protocol. The scintigraphic procedure includes 1 and 4 hours postinjection planar and SPECT images of the tumour regions. Tracer uptake was described as enhanced (+) or nonpathological (−).

### 2.2. Immunohistochemistry

After methodological setting by testing the SSTR 2A antibody (code SS-800, Biotrend Chemikalien, Cologne, Germany), all cases of desmoid tumors with available pathological material were retrospectively analyzed for immunohistochemical expression of SSTR 2A (Figure 1(b)).

All specimens were fixed in formalin, routinely processed and paraffin embedded. Immunohistochemical studies were performed using an established specific rabbit polyclonal antibody against SSTR 2A receptors (code SS-800, Biotrend Chemikalien), more precisely against its carboxyl-terminus (amino acid sequence 355–369 (ETQRTLLNGDLQTSI)).

The tissue sections were processed under the terms of manufacture guidelines or standard protocols. Appropriate positive and negative controls were included: specimens of neuroendocrine tumours (NETs) were stained as positive control, while normal human soft tissue was used as negative control. The staining pattern was described as absent (score 0: no immunoreactivity), moderate (score 1: focal to multifocal immunoreactivity in <50% of cells), and distinct (score 2: immunoreactivity in >50% of cells). All slides were evaluated independently by light microscopy by two of the authors, including a senior pathologist (B. Atzwanger).

## 3. Results

### 3.1. Somatostatin Receptor Scintigraphy

Ten patients were examined with SRS at least once and three were investigated twice. In all three cases, the findings of the first scintigraphy correlated with the findings of the second investigation (Table 1). Altogether, five cases showed nonpathological tracer uptake (38%), while eight cases (62%) revealed increased tracer uptake at tumour site and were therefore considered as SSTR expressing tumors (Figure 1(a)).

### 3.2. Immunohistochemistry: SSTR 2A

Tissue samples from seven patients were available and were exclusively specimens from extra-abdominal locations. In total, nine specimens were tested for the expression of SSTR 2A, seven tissue samples were from one location of the tumour and in two cases, specimens from two various locations of the tumour were investigated. Staining was moderate in four cases and absent in five cases (Figure 1(b)). Distinct staining was not found in any of the samples. In two cases, two tissue samples of two various locations of the tumour were tested. One of these samples stained neither in the first nor in the second location. The other sample did not stain in the first location, but revealed moderate staining in the second location.

### 3.3. Correlation of SRS and Immunohistochemistry

The findings of SRS and immunohistochemistry correlated in only two patients. In these cases the desmoids showed an adequate tracer uptake in SRS and their tumour samples revealed positive staining by immunohistochemistry (Figures 1(a) and 1(b)). Altogether, five cases showed inconsistent SRS results as compared with immunohistochemistry. Three of these had adequate tracer uptake in SRS, while the tissue samples revealed absent staining. On the other hand, in one patient the tumour showed non-pathological tracer uptake in SRS, while the tissue specimen stained moderately. Another patient showed non-pathological tracer uptake in SRS and the tissue sample revealed absent staining of one location and moderate staining of the other location. In conclusion, a positive correlation between SRS and immunohistochemistry could be found in two patients (29%).

## 4. Discussion

In the present investigation, 62% of the examined proliferations showed an enhanced tracer uptake at the site of the desmoid lesion in SRS, hence these tumors were regarded as SSTR expressing proliferations. Desmoids of five cases (38%) showed nonpathological scintigraphic findings and, consequently, were considered as SSTR negative lesions. Furthermore, three patients, who have been investigated twice, revealed correlating findings of the first and the second scintigraphy which is in agreement with the generally admitted fact that recurrences usually express the same receptors as the primary tumors and, additionally, show the same tracer uptake pattern.

Somatostatin receptors are frequently expressed in a wide variety of neoplastic proliferations with soft tissue tumors and desmoids being no exception [14]. Although the amount of SSTRs as well as the expressed subtypes are varying from tumour to tumour and within one tumour, SSTR 2 is the most frequently expressed subtype in the vast majority of SSTR positive tumors [23] and, moreover, its splice variant SSTR 2A has been shown to be the actually expressed isoform [24, 25]. This is of diagnostic importance for SRS, since commercially available radiopharmaceuticals contain the somatostatin analogue octreotide or derivates of the same and have distinct affinity to SSTR 2 and only moderate affinity to subtypes 3 and 5.

In 2003 data was published leading to the assumption that desmoid tumors may express SSTRs as well. Florio et al. [14] reported on four samples of aggressive fibromatosis and analyzed them for the expression of SSTRs by using RT-PCR (RNA isolation and reverse transcription-polymerase 44 chain reaction). They found that two samples where negative for all SSTR subtypes, while one abdominal fibromatosis expressed SSTR 1–4 and one extra-abdominal fibromatosis located on a limb expressed SSTR 1 and SSTR 5 [14]. In 2003, de Pas et al. [22] examined six patients with fast growing relapses of desmoid tumors using SRS and found that two of them were SSTR positive. Subsequently, they were apparently successfully treated (partial response or disease stabilization) with 90Y-DOTATOC.

Immunohistochemical evaluation of SSTR 2A in human tumors is well established and has been shown to be a fast and reliable method [24]. De Pas et al. [22] reported on ten patients with aggressive fibromatosis, among whom two showed immunopositivity to a rabbit polyclonal antibody SSTR 2A. Körner et al. [26] found that one antibody, namely the commercially available SSTR 2A antibody (code SS-800, Biotrend Chemikalien, Cologne, Germany), which has also been used in the current study, is suitable for routine immunohistochemical assessment of the SSTR 2A status of neoplastic tissues. The main disadvantage of immunohistochemical investigations of SSTRs is the general lack of standardization concerning interpretation of staining patterns, which results in problematic reproducibility of this method [27]. In this series, nine specimens have been tested for the expression of SSTR 2A. In our study only four cases revealed a moderate staining by immunohistochemistry whereas five cases were completely negative.

Several studies have directly compared the results of SRS with corresponding immunohistochemical SSTR 2A investigations and none of these concerned desmoid tumors [23, 27, 28]. Nevertheless, reasons for discrepant findings among these two techniques are presumably independent from the examined tumour type. As with this study, two various constellations of discrepant SRS and immunohistochemistry findings can occur: discrepancies between enhanced tracer uptake in SRS and absent immunohistochemical staining for SSTR 2A or nonpathological tracer uptake in SRS and positive immunohistochemical staining. For both constellations several explaining theoretical approaches exist.

First, the somatostatin analogues used for SRS binding, apart from SSTR 2, also the subtypes SSTR 3 and 5 with moderate affinity. Although SSTR 2 is the most frequent expressed receptor subtype in most tumors, it is possible that scintigraphy results are positive because of the other two receptor subtypes predominating within the lesion. However, since this survey investigated immunohistochemical expression of SSTR 2A only, no assertion can be stated, though some authors [23, 27] found that the analysis of the other two receptors did not increase the congruency of the two techniques.

Second, nonpathological tracer uptake in SRS can be based upon a poor SSTR density and may be another explanation for inconsistent results, since Asnacios et al. [28] reported that nonpathological scintigraphy combined with positive immunohistochemical staining implicate an overall lower amount of positive tumour cells as compared with correlative positive results of both techniques. In the current study, the amount of immunohistochemical stained cells in positive histological sections was lower than 50% in all samples and regardless of enhanced or non-pathological SRS. Degradation of SSTRs in tissue specimens with time may another reason for the inconsistent results between SRS and immunohistochemistry [23].

Another important factor might be the used antibody for immunohistochemical staining as well as the area of its binding. In an earlier series, our study group found positive staining for somatostatin in only six of 46 extra-abdominal, two of 21 abdominal, and one of 13 intra-abdominal fibromatoses (overall 11%) using a polyclonal, cytoplasmatic somatostatin antibody (code A0566, Dako, Vienna, Austria) [29]. In the current series, a surface antibody for SSTR 2A (code SS-800, Biotrend Chemikalien) was used being positive in four of nine cases (44%), indicating higher specificity.

Several authors [23, 27] agree that the frequently found heterogeneous distribution of SSTR subtypes in neoplasms may be the most plausible explanation for both, discrepant positive and discrepant negative results in immunohistochemistry as compared with SRS. In concordance with this assumption, the tissue sample of one patient with discrepant results had been obtained by biopsy, thus it is not representative for the SSTR expression pattern of the whole tumour. Moreover, except for two tissue samples, only one histological section per specimen has been examined for the expression of SSTR 2A, which again seems to be unrepresentative for the whole tumour. The tumors' frequently found heterogeneity is also emphasized by the tissue sample of one case that has been investigated on two various locations and showed opposed results.

Nevertheless, the appropriate reasons for the discrepant results remain elusive. The greatest problem seems to be the general lack of standardization of immunohistochemical SSTR analysis and, therefore, the development of standardized scoring systems is of prime importance. Furthermore, immunohistochemistry fails to provide information on the whole tumour and one histological section per tissue sample seems diagnostically inconclusive. Consequently, immunohistochemistry seems to provide the more convincing results, the more histological sections of different locations of one tumour have been investigated. However, the value of immunohistochemical SSTR 2A examinations of desmoid tumors remains unclear and further investigations are needed.

## 5. Conclusion

The current study demonstrated that desmoid tumors frequently express SSTR 2. Overall, eight out of 13 desmoids (62%) showed an enhanced tracer uptake. On the other hand, immunohistochemical investigations for SSTR 2A did not correlate with findings of somatostatin receptor scintigraphy. This may likely be due to the lack of standardization of this technique as well as the heterogeneous receptor distribution within the tumors. Nevertheless, further investigations are needed to determine the value of somatostatin receptor scintigraphy and immunohistochemistry for desmoid tumors.

## Figures and Tables

**Table 1:** Patient demographics, summarizing age, sex, type, and location of desmoid tumour, results of SRS (SRS was performed once (SRS 1) and occasionally a second time (SRS 2)) and immunohistochemistry and followup in months. Tracer uptake was described as + (enhanced) and − (nonpathological). SSTR 2A staining was described as 0 (absent), 1 (moderate), and 2 (distinct).

No.	Sex and age	Type, location and side	Diagnosis	SRS 1	SRS 2	SSTR 2A	Followup
1	M, 44	Extra-abdominal fibromatosis, upper arm, right	1st relapse	In^111^−	In^111^−	1	47
2	F, 52	Extra-abdominal fibromatosis, thigh, right	Primary tumor	In^111^+	In^111^+	n.a.	61
3	F, 35	Abdominal fibromatosis, abdomen, left	Primary tumor	In^111^−	n.a.	n.a.	19
4	F, 29	Extra-abdominal fibromatosis, shoulder, right	1st relapse	In^111^+	n.a.	0	134
5	M, 18	Extra-abdominal fibromatosis, lower limb, right	2nd relapse	In^111^+	n.a.	n.a.	153
6	F, 20	Extra-abdominal fibromatosis, upper limb, left	Primary tumor	In^111^+	n.a.	0/0	93
7	F, 48	Abdominal fibromatosis, abdomen, right	Primary tumor	In^111^−	n.a.	n.a.	6
8	M, 36	Extra-abdominal fibromatosis, thigh, left	1st relapse	In^111^+	n.a.	0	71
9	M, 33	Extra-abdominal fibromatosis, metacarpus, right	2nd relapse	In^111^−	n.a.	n.a.	14
10	F, 27	Extra-abdominal fibromatosis, planta pedis, right	2nd relapse	In^111^+	n.a.	1	54
11	M, 50	Extra-abdominal fibromatosis, elbow, right	1st relapse	Tc^99m^−	n.a.	0/1	27
12	M, 52	Extra-abdominal fibromatosis, shoulder, right	Residual tumor	Tc^99m^+	Tc^99m^+	1	15
13	F, 52	Extra-abdominal fibromatosis, thoracic, right	1st relapse	Tc^99m^+	n.a.	n.a.	13
